# Supplementary material for: Generalizability challenges of mortality risk prediction models: A retrospective analysis on a multi-center database
Source: PLOS Digit Health. 2022 Apr 5;1(4):e0000023. doi: 10.1371/journal.pdig.0000023 (PMC9931319; doi:10.1371/journal.pdig.0000023)
Supplement: Attachment — Submitted filename: Response to Reviewers.docx [file pdig.0000023.s002.docx]

We thank the reviewers for the thoughtful comments on the manuscript. We are pleased that they found the findings to be interesting and well-presented. Below are our point-by-point responses to all the issues raised that primarily involve elaborating the presented results (R1), and model development details (R2). The manuscript incorporates all the suggested changes. We would be happy to make further changes if any remain.

Reviewer 1

1. Training models from a given region/hospital and then testing those models on a different hospital/region is certainly a good way to explore generalizability. However, it may also be interesting to train on all of the data and then check to see how that generalizes to specific hospitals/regions. Was this approach considered as part of the study design?

- We based our study design on the highly practical setting where the models are trained on one collection of datasets and *transported* to another collection not used in the training, thus studying generalizability in the sense of external validity (or transportability). This precludes the setting (even though an important one) that the Reviewer mentions since, by design, all potential datasets are not available during training. Our setting uses hospital / region as our dataset collections to mirror the concerns that models trained only on a single health system or geography may introduce systemic biases or learn non-generalizable patterns (references 11, 76, in the manuscript).

2. In the S1 Table, for the elective surgery variable, in nine of the ten hospitals false is much more common than true but for Hospital ID 458 the opposite it true. Is the result shown for this hospital correct?

- This indeed is a typo. We mistakenly swapped numbers for True and False while formatting the table. We have corrected this. As is the case for the other nine hospitals, the elective surgery variable being false is more common than being true for ID 458 too. Thanks for making such a careful observation.

Correct numbers for ID 458 electivesurgery are True: 413 (19.6), False: 1689 (80.4).

3. In the S2 Table, the median age of patients is exactly the same across all four regions. Is this correct?

- We confirmed that the median (and the 25^th^-75^th^ percentile) age is the same across the four regions. The mean age does have small differences, as included below.

| **Region** | **Median [25^th^-75^th^ percentile]** | **Mean** |
| --- | --- | --- |
| Midwest | 52.0 [64.0, 75.0] | **61.90** |
| Northeast | 52.0 [64.0, 75.0] | **62.05** |
| South | 52.0 [64.0, 75.0] | **62.18** |
| West | 52.0 [64.0, 75.0] | **62.42** |

4. On line #245, the text mentions changes in AUC and CS but numerical results are only provided for AUC. I’d recommend showing the numerical result for CS also since it is mentioned in the text alongside AUC.

- We have included results for CS as well. The updated line reads as follows:

> Percentage changes in AUC and CS from train to test set, reported in Table S4 also indicate substantial drop in performance (in the range of -2.5% to -31.5% in AUC and -15.9% to -45.4% in CS).

5. On line #290, the text references Figure S3 but does not explain what it is showing. If a figure is referenced in the results section it should be accompanied by a brief explanation about what it is showing.

- Based on Reviewer’s suggestion, we have described Figure S3 briefly and made the connection between Figure S3 and Figure 4 clearer.

6. On line #308, the term “high-quality care” is used when discussing the direct effects of race on elective surgery. It isn’t clear to me that quality of care is directly related to whether or not elective surgery is chosen. Perhaps this should be explained further or another term should be used for the effect of race on elective surgery.

- We agree that access to “high-quality care” is not necessarily tied to availing elective surgery, and our intent for the term was to signify access to special procedures like surgeries. Thus, we have changed it to “specialized care”. Quoting from the referenced commentary (44):

> “… it has been shown that the hospitals that care for racial minorities may be limited in capability to perform needed interventions, like coronary artery bypass graft (CABG) surgery. (6)”

Thus, the use of specialized care is more apt.

7. Finally, I’d encourage double-checking the manuscript for grammatical errors. I noticed a number of sentences which were missing words.

- We thank the Reviewer for highlighting this. We have edited the manuscript for grammatical errors. This mainly includes re-phrasing the sentences in Author Summary and correcting missing articles (like the) in few of the sentences elsewhere.

Reviewer 2

In the introduction, the sentence in line 73 should be re-written for a clear understanding.

- The edited sentence reads as follows:

> Within the statistics literature are several methods for computing minimum sample size and other best practices for assessing external validity of clinical risk prediction models (7–9). However, challenges resulting from assessing sub-group-level performance and data-shifts are not explicitly considered in such guidance (10).

Did the authors consider other missing data imputation techniques for the model covariates?

The values of the covariates in the training dataset should be used as reference to apply the imputation mean in the test set, otherwise results might be biased. The authors mention that the mean is applied to data, in line 153, but do not specify this detail. The difference in mean and standard deviation between the train and the full datasets might be small and not cause an impact on the normalization results, but this should be checked.

- We checked results with three popular missing data imputation techniques, one of which is suggested by the Reviewer. The table below shows the median (and 1^st^-3^rd^ quartiles) for AUC, CS, and FNR under different imputation techniques. We observe that the results are similar to the ones reported in the manuscript, which are based on mean imputation from the full dataset.

| **Imputation method \ Metrics** | **Mean over all data (original results)** | **Mean over training data (suggested by R2)** | **Median over training data** | **Single imputation using decision trees (Ref. 77, Ch. 4)** |
| --- | --- | --- | --- | --- |
| AUC | **0.801**  **(0.777 to 0.832)** | **0.802**  **(0.777 to 0.832)** | 0.791  (0.766 to 0.819) | 0.807  (0.782 to 0.837) |
| CS | **0.853**  **(0.725 to 0.983)** | **0.853**  **(0.726 to 0.986)** | 0.833  (0.715 to 0.977) | 0.864  (0.714 to 1.001) |
| FNR | **0.092**  **(0.046 to 0.168)** | **0.094**  **(0.045 to 0.169)** | 0.094  (0.043 to 0.174) | 0.107  (0.052 to 0.186) |

The metrics differ by < 0.002 between imputation by mean from training versus full dataset (second and third columns). Thus, we have not changed the results in the manuscript. We have added a remark in the manuscript that different imputation methods did not change the conclusions.

To respond to the second part of the question, we had used mean and standard deviation for the *training* datasets (after imputation) to standardize the features, as suggested.

In line 166 correct “a CS”.

- Corrected.

In the paragraph correspondent to line 216, the authors seem to be describing “bootstrapping”.

- We performed resampling *without* replacement instead of bootstrapping which typically refers to resampling with replacement. Sampling without replacement avoids including data points more than once in the test sets. This in turn avoids an inflated estimate of model performance. In our case, most hospitals and regions had enough data points that we did not have to resort to sampling with replacement (bootstrapping) to create large enough random samples for training.

When referring to the variables such as the example "urineoutput" in the main text, the authors may write "urine output".

- Corrected urineoutput, temp, sysbp to their full forms.

Reviewer 3

We thank the Reviewer for encouraging remarks on the work related to presentation of the results and the emphasis on generalizability opposed to simply on internal validity of models.

Other changes

- Expanded the acronym “NSF” in Funding/Support disclosure.

- Removed Figures from the manuscript file.

- Added the following reference

> 77. Little RJA, Rubin DB. Statistical analysis with missing data. Third edit. Statistical Analysis with Missing Data. 2014. 1–381 p. (Wiley series in probability and statistics).

- Updated the following references with their published versions

> 14. Obermeyer Z, Powers B, Vogeli C, Mullainathan S. Dissecting racial bias in an algorithm used to manage the health of populations. Science (80- ). 2019;366(6464):447–53.

> 33. Jiang Y, Krishnan D, Mobahi H, Bengio S. Predicting the Generalization Gap in Deep Networks with Margin Distributions. In: International Conference on Learning Representations [Internet]. 2019. Available from: <https://openreview.net/forum?id=HJlQfnCqKX>

> 49. Reps JM, Kim C, Williams RD, Markus AF, Yang C, Duarte-Salles T, et al. Implementation of the COVID-19 Vulnerability Index Across an International Network of Health Care Data Sets: Collaborative External Validation Study. JMIR Med Inf [Internet]. 2021 Apr;9(4):e21547. Available from: <http://www.ncbi.nlm.nih.gov/pubmed/33661754>

> 56. Nestor B, McDermott MBA, Boag W, Berner G, Naumann T, Hughes MC, et al. Feature Robustness in Non-stationary Health Records: Caveats to Deployable Model Performance in Common Clinical Machine Learning Tasks. In: Doshi-Velez F, Fackler J, Jung K, Kale D, Ranganath R, Wallace B, et al., editors. Proceedings of the 4th Machine Learning for Healthcare Conference [Internet]. PMLR; 2019. p. 381–405. (Proceedings of Machine Learning Research; vol. 106). Available from: https://proceedings.mlr.press/v106/nestor19a.html

> 72. Gebru T, Morgenstern J, Vecchione B, Vaughan JW, Wallach H, III HD, et al. Datasheets for Datasets. Commun ACM [Internet]. 2021 Nov;64(12):86–92. Available from: https://doi.org/10.1145/3458723
