# Supplementary material for: Generalizability challenges of mortality risk prediction models: A retrospective analysis on a multi-center database
Source: PLOS Digit Health. 2022 Apr 5;1(4):e0000023. doi: 10.1371/journal.pdig.0000023 (PMC9931319; doi:10.1371/journal.pdig.0000023)
Supplement: Attachment — Submitted filename: Response to Reviewers.docx [file pdig.0000023.s003.docx]

We thank the editor for an encouraging assessment of the work and for the comment on the impact of skewed data. The revised manuscript incorporates the suggested edit. We would be happy to make further changes if any remain.

Editor

“The significant skew in the data might be a bit of an issue. Ideally, analyses might include either upsampling or downsampling.   Discussion of the potential impact of the skew - either in the discussion or as a limitation -  would strengthen this paper.”

- We have added the following text to the Discussion and Limitations pointing to the impact of the skewed data and possible strategies to reduce the impact.

> Further, the datasets have a skewed proportion of the minority population (as low as 3.2% for hospital ID 338, Table S1). This may negatively impact model performance for minority groups since the number of training samples might be insufficient to learn group-specific outcome characteristics, which is a recognized source of bias termed as representation bias (78). To reduce the impact of skewed data, algorithmic fairness literature proposes strategies such as reweighting data points for different groups based on their proportions or classification error (79-81). Efficacy of such strategies to decrease the disparity metrics reported in our results can be investigated in future work.

> Finally, we do not assess the effect of resampling data points on model performance to address issues of skewed mortality-class distribution or minority-majority group distribution.

Other changes

- Added Competing Interests Statement.

- Included list of legends for the Supporting Information file.

- Added the following four references.

> 78. Suresh H, Guttag J. A Framework for Understanding Sources of Harm throughout the Machine Learning Life Cycle. In: Equity and Access in Algorithms, Mechanisms, and Optimization [Internet]. New York, NY, USA: Association for Computing Machinery; 2021. (EAAMO ’21). Available from: https://doi.org/10.1145/3465416.3483305

> 79. Chen RJ, Chen TY, Lipkova J, Wang JJ, Williamson DFK, Lu MY, et al. Algorithm fairness in ai for medicine and healthcare. arXiv Prepr arXiv211000603. 2021

> 80. Krasanakis E, Spyromitros-Xioufis E, Papadopoulos S, Kompatsiaris Y. Adaptive Sensitive Reweighting to Mitigate Bias in Fairness-Aware Classification. In: Proceedings of the 2018 World Wide Web Conference [Internet]. Republic and Canton of Geneva, CHE: International World Wide Web Conferences Steering Committee; 2018. p. 853–862. (WWW ’18). Available from: https://doi.org/10.1145/3178876.3186133

> 81. Pfohl SR, Zhang H, Xu Y, Foryciarz A, Ghassemi M, Shah NH. A comparison of approaches to improve worst-case predictive model performance over patient subpopulations. arXiv Prepr arXiv210812250. 2021
